# Review of Journal of Cardiovascular Magnetic Resonance 2009

**DOI:** 10.1186/1532-429X-12-15

**Published:** 2010-03-19

**Authors:** DJ Pennell, DN Firmin, PJ Kilner, WJ Manning, RH Mohiaddin, S Neubauer, SK Prasad

**Affiliations:** 1 CMR Unit Royal Brompton Hospital, Sydney Street, London SW3 6NP UK. National Heart and Lung Institute, Imperial College, Exhibition Road, London, SW7 2AZ UK; 2 Departments of Medicine (Cardiovascular Division) and Radiology, Beth Israel Deaconess Medical Center 330 Brookline Avenue, Boston, MA 02215 USA. Harvard Medical School, 25 Shattuck Street Boston, MA 02115 USA; 3 Department of Cardiovascular Medicine, University of Oxford, John Radcliffe Hospital, Oxford OX3 9DU, UK

## Abstract

There were 56 articles published in the *Journal of Cardiovascular Magnetic Resonance *in 2009. The editors were impressed with the high quality of the submissions, of which our acceptance rate was about 40%. In accordance with open-access publishing, the articles go on-line as they are accepted with no collating of the articles into sections or special thematic issues. We have therefore chosen to briefly summarise the papers in this article for quick reference for our readers in broad areas of interest, which we feel will be useful to practitioners of cardiovascular magnetic resonance (CMR). In some cases where it is considered useful, the articles are also put into the wider context with a short narrative and recent CMR references. It has been a privilege to serve as the Editor of the JCMR this past year. I hope that you find the open-access system increases wider reading and citation of your papers, and that you will continue to send your quality manuscripts to JCMR for publication.

## Ventricular volumes, function and mass

### A dual propagation contours technique for semi-automated assessment of systolic and diastolic cardiac function

Despite the known accuracy and reproducibility of CMR for the measurement of ventricular volumes and mass based on manual contour delineation, given the effort required, there is great enthusiasm for improvement and especially automation of the analysis. Recent papers have focussed on accelerated imaging [1,2], right ventricular (RV) function [3], and diastolic function [4]. In this study, Feng described a novel approach to semi-automated segmentation of the left ventricle (LV) with the aim of more accurately derive ejection and filling rates from volume time curves [5]. They used manually drawn end-systolic and end-diastolic contours and their algorithm used both forward and backward propagation to identify the rest of the contours. In both normals and hypertensive patients, this approach was shown to more closely follow throughout the cardiac cycle the manually drawn contours than previously described approaches that use only forward propagation from one time point. The automated analysis time saving (vs the manual approach) was considerable with an analysis time reduction from 4 hours to just 7 minutes.

### Electrocardiographic (ECG) criteria for determining LV mass in young healthy men; data from the LARGE Heart study

Doubts remain over the use of the ECG in identifying those with LV hypertrophy (LVH), which is particularly marked in young individuals because of high prevalence of ECG criteria for LVH. Sohaib compared ECG criteria of LVH with CMR as a gold standard in 101 male army recruits [6]. The paired sensitivity and specificity values for 4 ECG indices of LVH showed poor accuracy and the authors concluded that they have little value in young fit males.

### Determinants of LV mass in obesity

This study examined a group of 38 obese individuals, without additional cardiovascular disease risk factors, compared to normal weight controls [7]. LV mass and volumes were assessed with CMR. From a large set of CMR and anthropometric measurements, the study sought to determine the main predictors of LV mass. These were found to be lean body mass, LV stroke volume and abdominal visceral fat mass. These findings help with our understanding of the determinants of LV hypertrophy in obesity.

### Myocardial tissue tagging with cardiovascular magnetic resonance

This article reviewed the current state and applications of myocardial tagging using CMR [8]. Emphasis is given to its role in detection in subclinical myocardial disease. There is considerable interest in this technique, both in humans and animals [9,10]. and also in related techniques such as strain encoding [11].

### Comparison of 2D and 3D calculation of LV torsion as circumferential-longitudinal shear angle using cardiovascular magnetic resonance tagging

This study compared LV torsion based on the circumferential - longitudinal shear angle using 2D and 3D quantification. Both methods were effective although the 2D method was faster [12].

### Effects of deep sedation or general anesthesia on cardiac function in mice undergoing CMR

This paper examined the effects of mild sedation, deep sedation and general anaesthesia (GA) on heart rate and LV ejection fraction (EF), using both CMR and echocardiography [13]. In normal mice, heart rate and LVEF decreased on deep sedation, and more so on GA, while there were no such differences in mice with chronic heart failure. This study demonstrates the significant impact of different sedation/anaesthesia regimes on cardiac function. It also suggests that experiments under deep sedation are feasible and an alternative to GA, yielding more accurate measurements of cardiac function.

## Flow evaluation and valve disease

### Three dimensional three component whole heart CMR velocity mapping: comparison of flow measurements from 3D and 2D acquisitions

Brix described the application of multidirectional time resolved 3D or 7D phase contrast velocity mapping CMR to provide comprehensive flow data with whole heart coverage [14]. The resulting data were used to derive quantitative flow data which are compared to more traditional higher spatial and temporal resolution 2D datasets. These methods and in particular the application for the assessment and analysis of whole heart flow are of interest because the acquisitions can be made without preplanning and the flow in any plane can be analysed retrospectively. This study showed that the volume flows measured from the 7D data compared well to the measurement from predefined 2D single velocity direction acquisitions. Multidirectional flow analysis may find a useful role in understanding the pathophysiology of abnormalities of the vascular system [15].

### Pulmonary intravascular blood volume changes through the cardiac cycle in healthy volunteers studied by CMR measurements of arterial and venous flow

In a novel attempt to measure the cyclic changes of the volume of blood contained in the pulmonary vessels and microvessels, Ugander used CMR phase contrast measurements of flow through the pulmonary trunk and the four pulmonary veins in 10 healthy subjects [16]. This carefully performed study contributes new information to the relatively sparse literature on the biomechanical properties of healthy human pulmonary vasculature.

### The effects of breath-holding on pulmonary regurgitation measured by CMR velocity mapping

Breath hold acquisitions, usually after expiration, are widely used in CMR. Measurement by phase contrast velocity mapping in patients with repaired tetralogy of Fallot by Johansson [17]. indicated that expiratory breath holding reduced pulmonary regurgitant fraction compared with held inspiration or free breathing. Altered airway pressure may be a contributory factor.

### Biventricular adaptation to volume overload in mice with aortic regurgitation

This study investigated the hypothesis that hypercholesterolaemia leads to aortic regurgitation due to lipid deposition and fibrosis of the aortic valve [18]. This was investigated in normal control and hypercholesterolaemic mice, using CMR to assess aortic regurgitation and LV volumes. The prevalence and severity of aortic regurgitation was significantly increased in hypercholesterolaemia, and aortic regurgitation led to LV remodelling in hypercholesterolaemic mice. This study establishes a link between hypercholesterolaemia and aortic regurgitation, suggesting that systematic human studies of this topic are warranted, and also points towards a prophylactic treatment of aortic regurgitation.

### CMR of quinticuspid aortic valve with aortic regurgitation and dilated ascending aorta

Bicuspid aortic valves are unusual, the quadricuspid form is rare and the quinticuspid form is exceptional [19]. Few readers are likely to have seen images of a quinticuspid aortic valve before publication of the remarkable images submitted by Meng.

### Aortic valve stenotic area calculation from phase contrast CMR: the importance of short echo time

CMR has been shown to underestimate aortic flow in turbulent high velocity jets due to intra-voxel dephasing. This study investigated the effect of decreasing intra-voxel dephasing by reducing the echo time (TE) on aortic valve area (AVA) estimates in patient with aortic valve stenosis [20]. The authors showed that agreement of CMR AVA with echocardiography improves with reduce TE to 1.5 ms. Further improvements or novel more robust CMR phase contrast methods are suggested to improve assessment of severity of moderate-to-severe aortic stenosis. This report emphasises the need for continuing optimisation of CMR techniques in the measurement and assessment of valve regurgitation [21,22]. and stenosis.

### Electrocardiographic (ECG) diagnosis of LVH in aortic valve disease: evaluation of ECG criteria by CMR

This study examined ECG indices of concentric and eccentric LVH in patients with aortic valve disease and in healthy subjects, calibrated against CMR measurements of cardiac volumes and mass [23]. Several ECG indices of hypertrophy were assessed, which all correlated with CMR parameters, but the Romhild-Estes score was the most powerful index. Concentric and eccentric LVH could be distinguished by differences in intrinsicoid deflection and ST-segment and T wave changes. This study adds to our knowledge on the relationship between electrical and anatomical indices of LV hypertrophy.

## Congenital & Pediatric

### Improved accuracy in flow mapping of congenital heart disease using stationary phantom technique

Miller addressed the issue of errors in derived measurements of flow that are attributable to background offset errors in CMR phase contrast velocity acquisitions [24]. They applied a static phantom correction approach, previously reported by Chernobelky [25]. to clinical CMR studies of flow in 31 patients with congenital heart disease. Although flow measurements across their population of patients showed relatively little mean error, they found errors for some individual patient studies were significant, but could be satisfactorily corrected by the subsequent use of static phantom acquisitions.

### Normal RV and LV volumes and myocardial mass in children measured by steady state free precession

The CMR study by Valsangiacomo Buechel of RV and LV volumes and mass in 50 children without known cardiovascular disease undergoing steady state free precession (SSFP) CMR made an important contribution to the literature by recording reference values relative to body surface area [26]. The paper complements and supports another recently reported study [27]. and adds to the growing literature on CMR in children [28,29].

### Feasibility of perfusion CMR in paediatric patients

Concerns regarding the effects of ionizing radiation make CMR study of myocardial perfusion a potentially attractive option, particularly for younger patients. Valsangiacomo Buechel reports the results of first pass adenosine stress CMR perfusion studies in 47 patients between 1 month and 18 years of age, most of whom had either Kawasaki disease, previous arterial switch or Ross operation [30]. General anaesthesia was used for 18 of the studies. Sixteen of the CMR perfusion studies were judged to be abnormal, giving a sensitivity of 87% (CI 52-97%) and a specificity of 95% (CI 79-99%) relative to the presence of corresponding angiographically demonstrated coronary stenosis.

## Pericardium and tumours

### CMR in pericardial diseases

This is an excellent comprehensive review of contemporary knowledge of how CMR can be used to study most common pericardial diseases [31]. The review is well illustrated and referenced. Strong points in favour of using CMR for imaging in pericardial disease are the integration of anatomic and functional information within a single examination; tissue characterization to determine inflammation and activity of the disease and the value of CMR to accurately assess the rats of the heart, particularly the myocardium, helpful in the differential diagnosis, which often remain a diagnostic challenge.

### Validation of CMR assessment of pericardial adipose tissue volume

Pericardial adipose tissue has been shown to be an independent predictor of coronary artery disease. In this study CMR-derived pericardial adipose tissue volume has been shown to be accurately reflected pericardial adipose tissue mass in ovine model [32]. The technique is ideally suited for future studies of the role of pericardial adipose tissue in cardiovascular risk prediction and disease in clinical practice.

### Dressler's syndrome demonstrated by late gadolinium enhancement (LGE) CMR

This interesting case report demonstrated global pericardial inflammation late post-myocardial infraction visualized by LGE on CMR [33]. This may be clinically useful when trying to establish a cause for atypical post-myocardial infarction pain without requiring exposure to invasive procedures of ionizing radiation.

### CMR diagnosis of cystic tumor of the atrioventricular node

CMR is well established for assessment of cardiac tumors, which may show characteristic signal behaviour with T1 and T2 weighted sequences, as well as response to gadolinium [34,35]. In this case report, LGE CMR was shown to be useful for characterizing a rare primary cystic tumor of the atrioventricular node of a patient with recent syncope and abnormal AV nodal function referred for CMR for a suspected atrial myxoma [36]. Cine SSFP CMR demonstrated a 2.5 cm hypointense mass at the AV node with prominent homogeneous LGE. At surgery, a multicystic tumor was identified.

## Cardiomyopathy

### CMR findings in patients with hypertrophic cardiomyopathy (HCM) and atrial fibrillation

CMR has made possible major clinical advances in the phenotyping [37]. and risk assessment [38]. of a broad range of cardiomyopathies and a recent JCMR review on arrhythmogenic right ventricular cardiomyopathy (ARVC) has been one of the most accessed recent articles [39]. The assessment of risk in HCM is challenging clinically, and scarring shown by LGE may play a useful role, even in phenotypically normal HCM gene carriers [40]. Atrial fibrillation in HCM carries an adverse prognosis, and early detection is important but challenging. In this study by Papavassiliu, patients with LGE were shown to be more likely to develop atrial fibrillation [41]. However, left atrial dilatation which is a conventional risk factor for atrial fibrillation was a stronger predictor, indicating that further work is needed in this area to tease out the interplay between scarring and ventricular compliance in the genesis of atrial fibrillation.

### Early manifestation of alteration in cardiac function in dystrophin deficient mdx mouse using 3D CMR tagging

Patients with Duchenne muscular dystrophy may develop dilated cardiomyopathy, even at young age. This study assessed the ability of CMR tagging to detect subtle alterations in LV function in a genetic model of Duchenne muscular dystrophy, the mdx mouse [42]. Compared to control mice, mdx showed an early increase in myocardial regional strain and torsion, and a decrease later in life. At all time points, global LV function measurements were similar between groups. This study suggests that CMR tagging may be a sensitive technique to detect subtle alterations in cardiac function in patients with this disorder.

### T2 quantification for improved detection of myocardial edema

T2 weighted CMR has been used in patients with myocarditis [43,44] and acute myocardial infarction, in which it is useful in helping to differentiate acute from chronic myocardial infarction. However, T2 weighted imaging is sensitive to coil sensitivity and motion. In a phantom study, Giri studied several methods of T2 mapping [45]. T2-prepared SSFP demonstrated greater accuracy in estimating T2 of phantoms as compared with fast spin echo approaches. The optimized method was then applied to healthy subjects and a swine model of acute infarction and small patient subgroup. The T2 of normal myocardium was found to be 52.2 +/- 3.4 ms. Importantly, the T2 maps were found to be insensitive to phased array coil sensitivity and cardiac motion, thereby providing potential for increased accuracy for the detection of myocardial edema.

### CMR in the diagnosis of acute heart transplant rejection: a review

In this review article, Butler reviews the role of CMR in the diagnosis of acute heart transplantation, including edema imaging and LGE [46]. Whilst CMR is not a mainstream clinical test in these circumstances, further research may establish a clearer clinical role in the future.

### Recovery of methamphetamine associated cardiomyopathy predicted by LGE CMR

Myocardial LGE is usually absent in cardiomyopathies which can recover, such as Takotsubo and myocardial siderosis [47], and when present LGE suggests a non-reversible course as in the cardiomyopathy associated with trastuzumab [48]. It is well established that certain medications can cause a cardiomyopathy, and in this report reversible ventricular dysfunction was associated with the use of methamphetamine [49]. LGE was absent in this case, and predicted recovery at 6 months after cessation of the drug insult.

### Cardiac injuries in blunt chest trauma; CMR of myocardial infarction after blunt chest trauma: a heartbreaking soccer-shot

The editors of JCMR were intrigued to receive two separate reports of myocardial damage attributable to external chest trauma within a week of each other. Marina Higuet showed evidence of a relatively extensive infarction involving septal, inferior and lateral regions of the LV in a 12 year old boy who had been run over 6 years previously [50]. In a second case, they reported evidence of rupture of the interventricular septum in a 45 year old man, investigated the day after he suffered compression in a collapsed trench. In both cases, it was suggested that sudden expansion of the RV and consequent tension on the moderator band may have contributed to the myocardial damage seen. If so, compression of the abdomen and its venous reservoir might also have contributed. The case reported by Hannibal Baccouche showed numerous focal patches of LGE in a 26 year old man after a high velocity impact to the chest while playing soccer [51]. Acute chest pain suffered by the young man soon after the trauma and angiographic evidence of proximal left anterior descending stenosis and more distal occlusion suggested traumatically induced coronary thrombosis and embolisation.

### The efficacy of iron chelator regimes in reducing cardiac and hepatic iron in patients with thalassaemia major: a clinical observational study

A major success story for CMR has been the demonstration that quantification of cardiac iron through the use of myocardial relaxation measurements. T2* has in general been preferred to T2 (both are feasible) because of ease of acquisition [52,53]. Cardiac iron assessment allows the identification of patients of high risk of developing toxic cardiomyopathy that can be averted by early intensive iron chelation treatment. This is associated with a 71% reduction in cardiac mortality in beta-thalassemia major [54]. which is the most prevalent condition worldwide associated with transfusion dependency. Now thrown into sharp focus is the relative efficacy of the 3 clinically available iron chelators in removing cardiac iron and preventing the onset of heart failure [55]. Berdoukas used paired T2* CMR scans in 232 patients to compare chelation regimes in a non-randomised analysis, which is not rigorous but can give an indication of differences in clinical cardiac efficacy [56]. The combination treatment with both deferoxamine and deferiprone was the most effective treatment for the heart, whilst monotherapy with deferiprone or deferasirox were preferentially effective in the heart and liver respectively. Randomised comparisons of the 2 oral iron chelators would be welcome, but face significant hurdles in funding.

### Saw-tooth cardiomyopathy

This is a very unusual case of cardiomyopathy in a young infant. Left ventricular dysplasia and inward sawtooth like projections were present [57]. The clinical profile and implications are discussed.

### CMR findings in a case of Danon disease

Danon disease is a rare X-linked dominant, lysosomal glycogen storage caused by LAMP-2 protein deficiency which is usually diagnosed using immunofluorescence in striated muscle. It is known to cause a HCM phenotype. In this rare patient, very marked and widespread LGE was present which was largely absent in the septum, which would be unusual for HCM [58]. The diagnosis should be considered in males with coexisting mental retardation/learning difficulties, skeletal myopathy or muscle weakness. Mental retardation or skeletal myopathy can be present in female carriers, but less commonly than in affected males.

## Atheroma and vascular

### Dual stack black blood carotid artery CMR at 3T: Application to wall thickness visualization

The authors proposed a novel approach for rapid imaging of large sections of the carotid artery wall at isotropic spatial resolution [59]. Using an interleave acquisition of two 3D stacks with proposed motion sensitized segmented steady-state black blood gradient echo technique, coverage of the carotid artery trees on both sides was achieved in reasonable time. Application of this technique in 10 patients showed that thickening of the vessel wall can be identified and the suspicious segments can be targeted for subsequent high-resolution CMR.

### High resolution carotid black-blood 3T CMR with parallel imaging and dedicated 4-channel surface coils

Saam and colleagues presented a study that demonstrated the applicability of carotid CMR at 3T [60]. In their study the group employed a 4 channel surface coil and used this to demonstrate a good combination of signal to noise, pixel size and scan time for a multi sequence carotid imaging protocol.

### Prediction of coronary artery disease by a systemic atherosclerosis score index derived from whole-body MR angiography (MRA)

Whole body MRA catches the imagination and is used in clinical practice [61]. There is an association between extra-cardiac and coronary atherosclerosis, although this is variable [62]. This study provided important new data on this association [63]. Whole body MRA derived systemic sclerosis score index was shown not only associated with established cardiovascular risk scores but is also predictive of significant coronary artery disease. This suggests potential prognostic implications of this approach and underlines the importance of screening for coronary artery disease in patients with extra-cardiac manifestations of atherosclerosis.

### CMR parameters of atherosclerotic plaque burden improve discrimination of prior major adverse cardiovascular events

The prevalence and burden of aortic and carotid atherosclerosis in patients with prior major cardiovascular and cerebrovascular events (MACE) was examined in a black blood CMR study of 195 subjects [64]. Patients with a MACE history had higher plaque burden (wall thickness, wall area, and normalized wall area). After adjustment for age, sex, and traditional risk factors, carotid artery plaque eccentricity was also associated with prior MACE.

### The added value of longitudinal black-blood CMR angiography in the cross sectional identification of carotid atherosclerotic ulceration

This study showed that additional value of longitudinal black-blood CMR angiography to multislice cross-sectional CMR images for assessing carotid artery disease as it increases accuracy in the identification of carotid atherosclerotic ulceration [65].

### Effect of rosiglitazone on progression of atherosclerosis: insights using 3D carotid CMR

There has been evidence suggesting that rosiglitazone increases death from cardiovascular causes, but this is controversial. Varghese performed a randomized, placebo-controlled, double-blind study to evaluate the effect of rosiglitazone treatment on carotid atherosclerosis in subjects with type 2 diabetes with a primary endpoint of the change from baseline in carotid arterial wall volume, reflecting plaque burden, as measured by CMR [66]. There were no significant differences between groups after 1 year of treatment which did not suggest that progression of atheroma was a likely cause for the possible excess mortality seen with rosiglitazone, and therefore other mechanisms should be examined.

### CMR in carotid atherosclerotic disease

This is an excellent review of techniques and clinical applications of CMR in carotid atherosclerotic disease by a well known group with extensive experience in this field [67]. The review is well illustrated with comprehensive list of references and provides state of the art summary of current knowledge in this area.

## Coronary artery disease

### Appearance of microvascular obstruction on high resolution first-pass perfusion, early and LGE CMR in patients with acute myocardial infarction

Mather used their high resolution perfusion imaging sequence to investigate the important question regarding how well the conventionally used LGE methods can assess the extent of microvascular obstruction (MO) [68]. The high resolution perfusion sequence used a spatio-temporal undersampling method, k-t SENSE to accelerate the data acquisition by a factor of 7 times. The results showed that in patients with recent acute myocardial infarction, successfully treated with percutaneous coronary intervention, high-resolution first-pass perfusion CMR identified more cases of microvascular obstruction than either early or LGE images and that the extent of microvascular obstruction also appears larger on the perfusion scans. In their conclusion they postulated that first-pass perfusion imaging was likely to have given the most accurate assessment of MO, as it was not confounded by the diffusion of gadolinium into the no-reflow zone over time, a phenomenon that would lead to an underestimation of microvascular obstruction by any LGE, regardless of timing.

### Comparison of wall thickening and ejection fraction by CMR and echocardiography in acute myocardial infarction

LV function and wall motion are routinely assessed post percutaneous coronary intervention in evaluating the success of the technique, and using both echo or CMR [69]. The study shows that both techniques have limitations, particularly at the early stage post infarction, although these are less marked by 4 months.

### Infarct evolution in man studied in patients with first-time coronary occlusion in comparison to different species - implications for assessment of myocardial salvage

Scar and ischemic burden are now readily measured in humans using CMR [70]. Although the time course of infarct evolution (how fast myocardial infarction develops during coronary artery occlusion), is well known for several species, evidence in humans is limited. Hedström recruited 16 patients with acute myocardial infarction and compared the myocardium at risk by SPECT and T2-weighted CMR with ultimate infarct size measured by LGE [71]. The time to reach 50% infarction of the myocardium at risk was 288 minutes, which is significantly longer than seen in pigs, rats and dogs. Whilst this study has limitations, it is provocative and suggests that further work is needed in this area to determine

### Prognostic value of adenosine stress CMR in patients with low-risk chest pain

Perfusion CMR is rapidly entering clinical practice in many centres around the world [72]. and is easily combined with other CMR techniques in a comprehensive assessment protocol [73]. There is considerable promise in new techniques [74], including acquisition acceleration [75], post-exercise imaging [76], and the use of 3T [77-79]. In this study, perfusion CMR is used in the emergency setting, where approximately 5% of patients with an acute coronary syndrome are discharged from the emergency room with an erroneous diagnosis of non-cardiac chest pain. This study evaluated the negative prognostic value of adenosine perfusion CMR among 103 low-risk acute chest pain patients [80]. Patients were followed for a mean of 277 days for a combined endpoint of cardiac death, nonfatal acute myocardial infarction, re-hospitalization for chest pain, obstructive coronary artery disease (> 50% coronary stenosis on invasive angiography) or coronary revascularization. Adenosine perfusion CMR was negative in 89 patients (86.4%), and none of these patients reached the primary endpoint. The authors conclude that adenosine perfusion CMR holds promise as a useful tool to rule out significant coronary artery disease in patients with low-risk chest pain, as patients with negative adenosine stress CMR have an excellent short prognosis.

### Variability of myocardial perfusion dark rim Gibbs artifacts due to sub-pixel shifts

Ferreira and colleagues published a technical note describing a subtle feature of the Gibbs ringing dark rim perfusion artifact that had not previously been discussed [81]. The authors showed that the extent of the artifact was dependant on the exact position of the blood myocardial border of the heart with respect to the imaging pixels. This could explain some of the unpredictability of dark rim artifacts from subject to subjects and even in the same subject over the acquisition.

### Adverse effect of increased LV wall thickness on five year outcomes of patients with negative dobutamine stress

Dobutamine stress CMR is being used in many centres and some new approaches have been reported [82]. although it remains less popular than perfusion CMR. The 5-year prognostic value of LVH in patients with a negative dobutamine/atropine SSFP CMR stress was assessed in a 175 patient study of patients with a normal LVEF (> 55%) and no inducible wall motion abnormality [83]. In a multivariate analysis, wall thickness ≥ 12 mm was associated independently with an increase of cardiac death and myocardial infarction (OR 6.0, p = 0.002) and the combined end-point of cardiac death, myocardial infarction, unstable angina, and congestive heart failure warranting hospitalization (HR 3.0, p = 0.0005).

### Quantification of LGE CMR in viability assessment in chronic ischemic heart disease: a comparison to functional outcome

Scar volume assessed by LGE CMR is known to predict future cardiac events [84]. The prediction of recovery of LV systolic function after revascularisation can be made in several ways [85]. but the use of LGE CMR to assess residual viability in association with scar is now in widespread clinical use. There has been considerable debate over the best quantitative technique to measure the extent of LGE, either in absolute terms or in relation to LV mass. This is not only more rigorous than qualitative assessment by scoring in the setting of clinical trials, but is more reproducible. This study evaluated the quantification of LGE in relation to the clinical standard of viability, namely functional outcome 6 months after revascularization [86]. LGE was quantified by thresholding window setting at 2-8 SD above mean signal intensity of a remote normal region, and according to the full width at half maximum method. Dysfunctional segments were divided in 5 groups according to segmental extent of enhancement. The quantification methods had a strong influence on the total infarct size, but although 6SD was the best in predicting segmental functional outcome after revascularization, the difference with other methods was small and non-significant. Thus the authors argue that whilst the quantification technique is important for quantification of infarct extent, the various techniques are similar for prediction of regional viability.

### Cell tracking and therapy evaluation of bone marrow monocytes and stromal cells using SPECT and CMR in a canine model of myocardial infarction

This study establishes a new large animal model suitable for the assessment of stem cell therapy for myocardial infarction [87]. Autologous bone marrow monocytes and stromal cells were used, and cell imaging achieved with 111Indium-tropolone labelling. LGE CMR was used to determine infarct shrinking. Initial results in dogs studied for 12 weeks following a 3 hour coronary occlusion show that monocytes produce significant treatment effects. This new multi-modality approach should be useful for the evaluation of new stem cell approaches to treating myocardial infarction.

### Reduced peripheral arterial blood flow with preserved cardiac output during submaximal bicycle exercise in elderly heart failure

This study addressed the problem that older patients with heart failure typically show exercise intolerance [88]. This could be due to lower blood flow to the lower extremities due to reduced cardiac output, or due to other mechanisms. Patients and controls were studied with CMR to measure aortic and lower extremities blood flow, and peak oxygen consumption (VO2) was also measured. In heart failure patients, lower extremities blood flow after exercise was indeed reduced, but aortic flow was not. This shows that mechanisms other than reduced cardiac output contribute to reduced exercise tolerance in heart failure in the elderly. The study demonstrates that exercise intolerance in heart failure in the elderly isn't simply a result of cardiac dysfunction, which may have implications for its optimal treatment.

## Electrophysiology and interventional

### CMR guided electrophysiology studies

The role of CMR guided electrophysiology studies was addressed in a review by the Johns Hopkins group [89]. Pre-procedure CMR was noted to improve the understanding of the anatomic basis of complex arrhythmias with a unique advantage of CMR identified as its ability to visualize prior ablation lesions using high-resolution LGE methods. Such information could then be applied to assess ablation lesion location and the potential identification of factors leading to procedure success and failure. The ability to perform intra-procedure real-time CMR to identify complex 3D arrhythmogenic anatomy and to target additional ablation areas was identified as future initiatives along with the requirement of clinical grade CMR compatible electrophysiologic devices. This review complements another recently published JCMR review on interventional CMR [90].

### Radial dyssynchrony assessed by CMR in relation to LV function, myocardial scarring and QRS duration in patients with heart failure

Mechanical dyssynchrony is a hot topic in heart failure, because of problems in predicting response to cardiac resynchronisation therapy (CRT). There is also debate regarding which non-mortality endpoint is best for assessing a good response to CRT [91]. CMR has been proposed be useful in this field [92]. and increasing interest in its use is being seen [93]. Radial dyssynchrony was found to be highly prevalent in a CMR tissue synchronization index (CMR-TSI) study of 225 patients with heart failure and 50 healthy controls [94]. Using a dyssynchrony threshold of healthy control mean TSI ± 2SD, dyssynchrony was found in 91% of subjects with a QRS duration < 120 ms, 95% of those with a QRS 120-149 ms, and 99% of subjects with a QRS ≥ 150 ms. CMR-TSI was related positively to LV volumes and inversely with LV ejection fraction.

### CMR Visualization of coronary venous anatomy by

Combined whole heart coronary vein MRA and LGE for patients with heart failure in whom cardiac resynchronization is being considered was retrospectively assessed in 31 subjects with known or suspected ischemic disease [95]. Within 5 minutes after administration of 0.05 mmol/kg Gd-DTPA, whole heart coronary vein MRA demonstrated 74% of subjects having a lateral vein, 65% an anterior interventricular vein, and 74% a posterior interventricular vein. Two patients had no venous branches other than the coronary sinus and great cardiac vein. An additional 0.15 mmol/kg of Gd-DTPA was then administered with LGE imaging 10-15 minutes later with 5 subjects having evidence of LGE. Co-localization of coronary vein imaging and LGE data may allow for improved efficacy of cardiac resynchronization therapy.

### Whole shaft visibility and mechanical performance for active MR catheters using copper-nitinol braided polymer tubes

Kocaturk published the development of a new active catheter design incorporating in its shaft a wire lattice in a polymer matrix [96]. The catheter contained three distal loop coils in a flexible and torquable 7Fr device. The group explored the impact of braid material designs on radiofrequency and mechanical performance. They found a 16-wire braid of 1:1 copper:nitinol to have the optimum balance of mechanical and antenna properties. With that configuration, the temperature increase remained less than 2°C during real-time CMR and the catheter was shown to be conspicuous *in vitro *and *in vivo*.

## New techniques

### Diffusion CMR tractography of the heart

Sosnovik and colleagues presented an extremely thorough review of diffusion encoding of the myocardium [97]. Much of the manuscript is based on a recent publication of the authors and as such it focused on diffusion spectral imaging (DSI) with application to tractography of *ex vivo *animal hearts. More conventional encoding approaches utilizing fewer diffusion encoding directions and b values are summarized particularly with regard to *in vivo *human imaging.

### CMR Elastography: Comparison with LV pressure measurement

Elgeti and colleagues presented early pilot data on the application of CMR elastography to studying the heart [98]. When further developed and validated these methods could have a major impact on clinical CMR. In this limited study on three pigs the authors demonstrated temporally within the cardiac cycle an earlier change in measured shear wave amplitude than the change in the LV diameters. The shear wave measurements also appeared to be related to the intraventricular pressure.

## SCMR papers and Registry

### Society for Cardiovascular Magnetic Resonance (SCMR) guidelines for reporting CMR examinations

Ongoing efforts for standardization have recently included CMR imaging protocols [99]. This has now been extended to CMR reporting with publication of SCMR report guidelines as an effort to provide a framework for healthcare delivery systems to disseminate cardiac and vascular CMR findings [100]. The SCMR recommends reporting key elements in all documents including information pertaining to a) site and equipment, b) patient demographics, c) study indications, d) study performance, e) cardiovascular imaging features of the examination, and f) concluding statements that synthesize the study results into a comprehensive diagnosis that can be used for planning therapy or determining prognosis. The document is comprehensive in scope and includes recommended and optional components of the report, the principles used to generate the final report, and suggested communications that may occur in addition to the final report.

### Current variables, definitions and endpoints of the European Cardiovascular Magnetic Resonance Registry

This paper describes the European Cardiovascular Magnetic Resonance Registry [101]. This multicentre registry will serve two functions: record the details of clinical CMR scans in all participating centres; follow patients with HCM and with coronary artery disease up annually to obtain prognostic data. This database, once available in several years, will serve many important functions - it will inform about the practice of CMR in Europe and its clinical utility, and it will yield important large scale prognostic information on CMR parameters obtained in patients with HCM and coronary artery disease.

### SCMR President's Pages

Chris Kramer was President of the SCMR in 2009 and wrote 2 Presidential Pages for the Journal. In the first, he reviewed the 2009 Annual Scientific Sessions which maintained attendance at over 1000 despite the economic downturn, and had 100 oral abstracts and over 300 poster presentations [102]. Membership was reported as stable at 1685 with total assets at the end of year 2008 are up 17% from prior year. The contributions of SCMR were highlighted in the drive to establish reimbursement for CMR payment codes, Appropriate Use Criteria and the Medical Imaging Drugs Advisory Committee of the FDA. In addition, the flourishing roles of the regional chapters, the SCMR web site (500,000 unique hits) and the many SCMR committees were detailed. In the second Presidential page, Chris was able to report the improved impact factor for Journal from 1.87 to 2.15, an increase of 15% [103], and this may reflect the first signs of increased citation associated with Open Access publishing [104]. He also detailed plans for an SCMR designed research trial which was in the late stages of planning, and for an update on training and credentialing, including technologists who perform CMR.

## Competing interests

DJP is a consultant to Siemens, Novartis and Apotex. DJP receives research support from Siemens and Novartis. DJP has received speakers honoraria from Siemens, Novartis and Apotex. DNF and SN receive research support from Siemens. SKP, PJK WJM and RHM declare that they have no competing interests.

## Authors' contributions

All authors contributed summaries of articles to the text. DJP wrote the final draft. All authors read and approved the final manuscript.
